# Supplementary material for: Temperature-dependent responses of the hard corals Acropora sp. and Pocillopora verrucosa to molecular hydrogen
Source: PLoS One. 2025 Mar 25;20(3):e0308894. doi: 10.1371/journal.pone.0308894 (PMC11936180; doi:10.1371/journal.pone.0308894)
Supplement: S1 File — (PDF) [file pone.0308894.s001.pdf]

Supporting Information

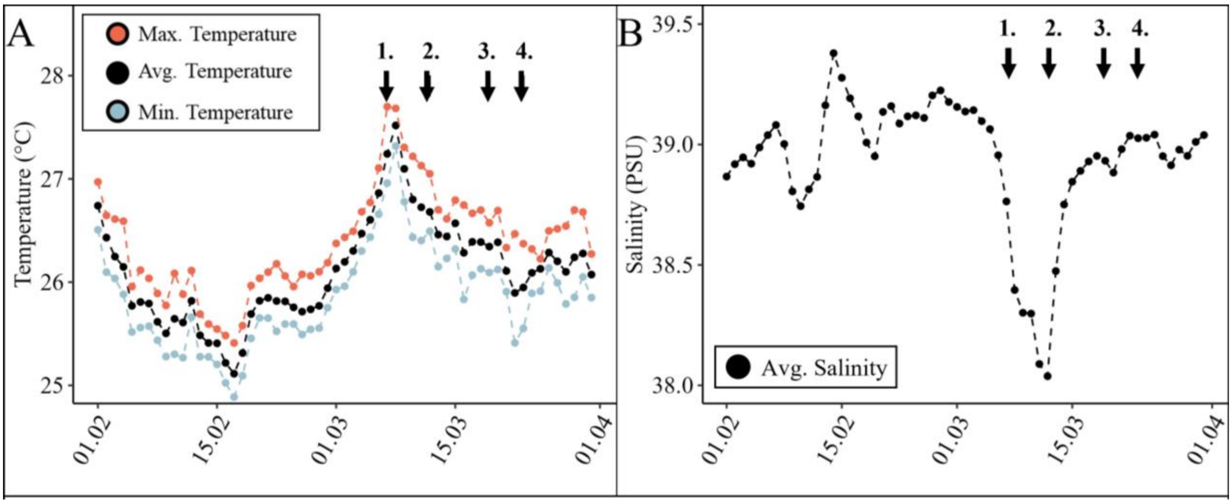

Fig. S1 Seawater temperature (A) and salinity (B) from 01st of February to the 01st of April 2023. The average daily seawater temperature (°C) and salinity (PSU) are indicated by black dots and the maximum and minimum daily temperature by red and blue dots, respectively. Bold arrows with numbers represent the four different sampling days.

Table S1 Precise seawater temperatures and salinities for the four different sampling days. The table below displays the precise average, minimum and maximum temperature and average salinity values on each sampling day relating to the four groups. The sampling identification represents the numbered bold arrows from Fig. S1.

**Sampling ID**	**Date**	**Group**	**Avg. Temperature [°C]**	**Min. Temperature [°C]**	**Max. Temperature [°C]**	**Avg. Salinity [PSU]**
1	07.03.23	$H_2$26	27.24	26.96	27.70	38.76
2	12.03.23	CT32	26.68	26.49	27.05	38.04
3	19.03.23	$H_2$32	26.34	26.09	26.58	38.93
4	23.03.23	CT26	25.95	25.55	26.37	39.03

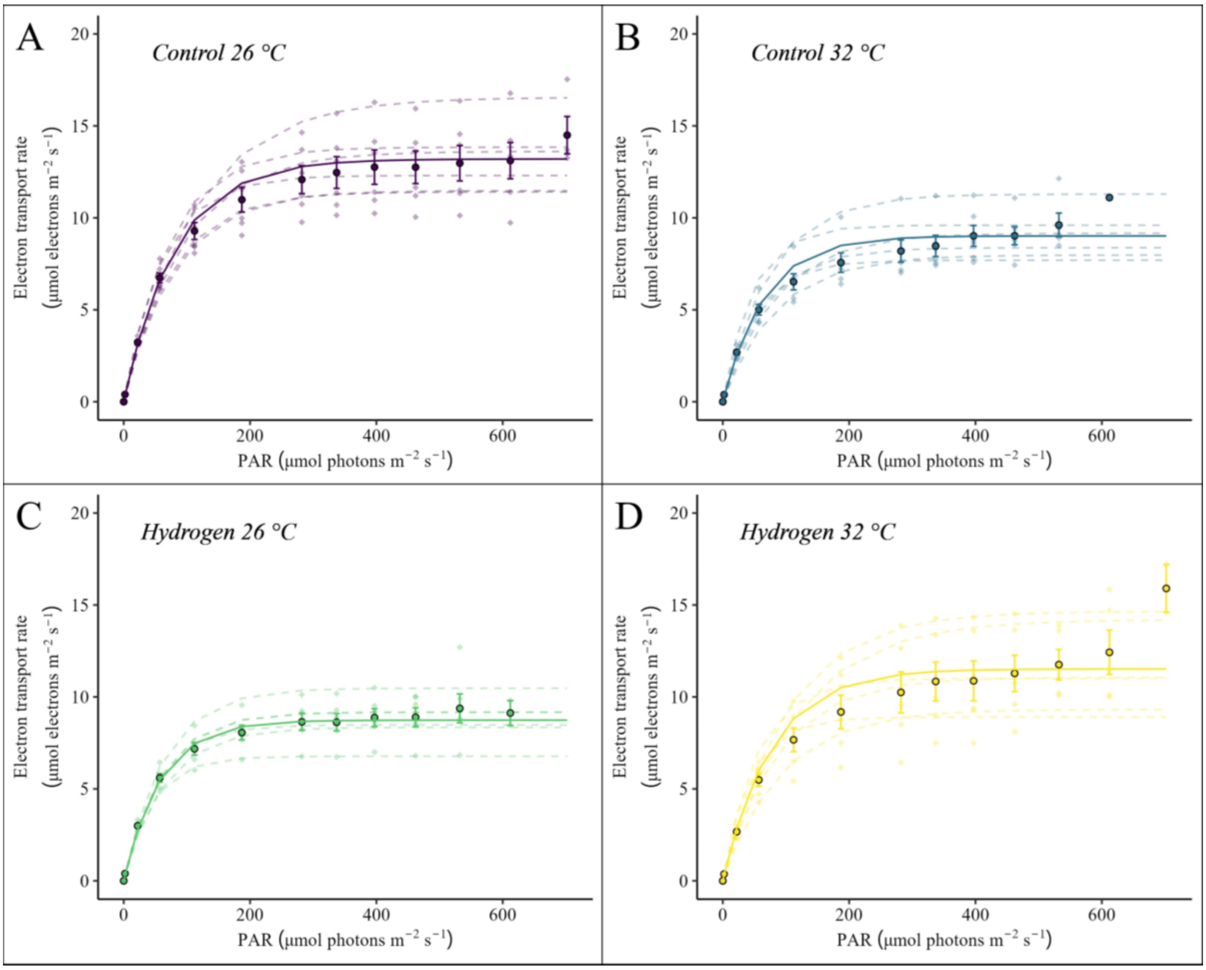

Fig. S2 Rapid light curves derived from the model fitted according to Platt et al. [1] for all four groups belonging to *Acropora* sp.. All six rapid light curves and respective mean values per group originating from the five rapid light curves per fragment are shown in pale colors for group CT26 (A), CT32(B), $H_2$26 (C) and $H_2$32 (D). In addition, an average rapid light curve and its respective mean values with standard error (SE) obtained from the six rapid light curves per group is represented in dark color with points and error bar.

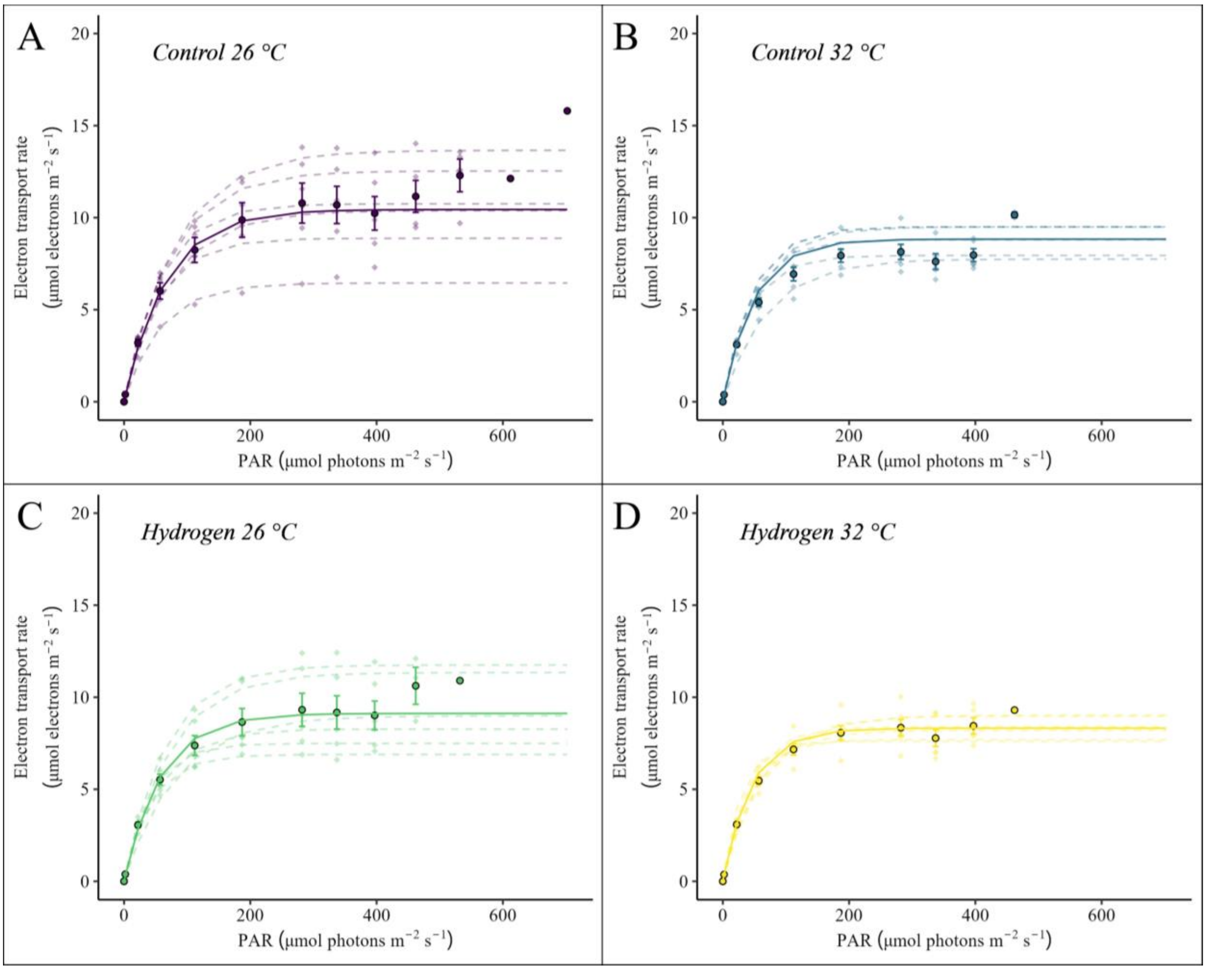

Fig. S3 Rapid light curves derived from the model fitted according to Platt et al. [1] for all four groups belonging to *Pocillopora verrucosa*. All six rapid light curves and respective mean values per group originating from the five rapid light curves per fragment are shown in pale colors for group CT26 (A), CT32(B), $H_2$26 (C) and $H_2$32 (D). In addition, an average rapid light curve and its respective mean values with standard error (SE) obtained from the six rapid light curves per group is represented in dark color with points and error bar.

Table S2 Results of the two-way PERMANOVA (type III) and Monte-Carlo test for the photosynthesis and respiration rates of *Acropora* sp.. Two factors including temperature (T) and treatment (Tr) as well as the interaction effect between temperature and treatment (T x Tr) were tested for significance in the two-way PERMANOVA. P-values in bold indicate a significant effect (p < 0.05).

Group	Variable	Factor	*df*	SS	MS	Pseudo-F	P (perm)	Unique	P (MC)
Oxygen Fluxes	Net Photosynthesis (P_{net})	Temperature (T)	1	0.04	0.04	0.04	0.843	997	0.824
		Treatment (Tr)	1	0.41	0.41	0.40	0.527	995	0.536
		T x Tr	1	2.35	2.35	2.29	0.148	997	0.157
		Residuals	18	18.43	1.02				
		Total	21	21.00					
	Respiration (R)	Temperature (T)	1	6.95	6.95	10.26	**0.008**	994	**0.004**
		Treatment (Tr)	1	0.06	0.06	0.09	0.738	997	0.748
		T x Tr	1	1.12	1.12	1.66	0.232	996	0.189
		Residuals	18	12.19	0.68				
		Total	21	21.00					
	Gross Photosynthesis (P_{gross})	Temperature (T)	1	3.37	3.37	4.23	**0.050**	998	**0.049**
		Treatment (Tr)	1	0.02	0.02	0.03	0.867	997	0.862
		T x Tr	1	2.53	2.53	3.18	0.103	998	0.091
		Residuals	18	14.33	0.80				
		Total	21	21.00					

Table S3 Results of the two-way PERMANOVA (type III) and Monte-Carlo test for the photosynthetic efficiency and variables derived from the rapid light curves (alpha, ps/ETR_{max}, E_k) of *Acropora* sp.. Two factors including temperature (T) and treatment (Tr) as well as the interaction effect between temperature and treatment (T x Tr) were tested for significance. P-values in bold indicate a significant effect ($p < 0.05$).

Group	Variable	Factor	*df*	SS	MS	Pseudo-F	P (perm)	Unique	P (MC)
Photosynthetic Efficiency	*Fv / Fm*	Temperature (T)	1	8.62	8.62	13.05	**0.001**	997	**0.003**
		Treatment (Tr)	1	0.63	0.63	0.95	0.343	996	0.337
		T x Tr	1	0.54	0.54	0.81	0.399	994	0.388
		Residuals	20	13.22	0.66				
		Total	23	23.00					
Rapid Light Curves	alpha	Temperature (T)	1	1.14	1.14	1.09	0.310	998	0.312
		Treatment (Tr)	1	0.00	0.00	0.00	0.994	996	0.997
		T x Tr	1	0.89	0.89	0.84	0.384	998	0.351
		Residuals	20	20.98	1.05				
		Total	23	23.00					
	ps / ETR_{max}	Temperature (T)	1	0.46	0.46	0.91	0.361	991	0.358
		Treatment (Tr)	1	0.91	0.91	1.79	0.229	997	0.198
		T x Tr	1	11.49	11.49	22.68	**0.001**	998	**0.002**
		Residuals	20	10.14	0.51				
		Total	23	23.00					
	E_k	Temperature (T)	1	0.41	0.41	0.49	0.485	996	0.479
		Treatment (Tr)	1	0.44	0.44	0.53	0.462	998	0.486
		T x Tr	1	5.47	5.47	6.56	**0.021**	997	**0.019**
		Residuals	20	16.68	0.83				
		Total	23	23.00					

Table S4 Results of the two-way PERMANOVA (type III) and Monte-Carlo test for Symbiodiniaceae cell density and chlorophyll a and c2 content of *Acropora* sp. Two factors including temperature (T) and treatment (Tr) as well as the interaction effect between temperature and treatment (T x Tr) were tested for significance. P-values in bold indicate a significant effect ($p < 0.05$).

Group	Variable	Factor	*df*	SS	MS	Pseudo-F	P (perm)	Unique	P (MC)
Symbiodiniaceae Cell Density	Symbiodiniaceae Cell Density	Temperature (T)	1	3.92	3.92	4.71	**0.017**	999	**0.047**
		Treatment (Tr)	1	0.95	0.95	1.14	0.350	997	0.282
		T x Tr	1	1.49	1.49	1.79	0.202	999	0.194
		Residuals	20	16.64	0.83				
		Total	23	23.00					
Chlorophyll	Chlorophyll a ($\mu g\ cm^{-2}$)	Temperature (T)	1	1.79	1.79	1.99	0.159	996	0.154
		Treatment (Tr)	1	2.87	2.87	3.20	0.100	999	0.096
		T x Tr	1	0.40	0.40	0.44	0.528	997	0.494
		Residuals	20	17.94	0.90				
		Total	23	23.00					
	Chlorophyll a ($pg\ cell^{-1}$)	Temperature (T)	1	1.23	1.23	1.23	0.265	994	0.276
		Treatment (Tr)	1	1.63	1.63	1.62	0.222	996	0.189
		T x Tr	1	0.06	0.06	0.06	0.799	998	0.831
		Residuals	20	20.08	1.00				
		Total	23	23.00					
	Chlorophyll c2 ($\mu g\ cm^{-2}$)	Temperature (T)	1	0.57	0.57	0.53	0.504	993	0.455
		Treatment (Tr)	1	0.94	0.94	0.88	0.393	997	0.359
		T x Tr	1	0.00	0.00	0.00	0.948	997	0.958
		Residuals	20	21.49	1.07				
		Total	23	23.00					
	Chlorophyll c2 ($pg\ cell^{-1}$)	Temperature (T)	1	1.26	1.26	1.26	0.289	996	0.283
		Treatment (Tr)	1	1.56	1.56	1.57	0.218	996	0.231
		T x Tr	1	0.24	0.24	0.24	0.638	998	0.638
		Residuals	20	19.94	1.00				
		Total	23	23.00					

Table S5 Results of the two-way PERMANOVA (type III) and Monte-Carlo test for the photosynthesis and respiration rates of *Pocillopora verrucosa*. Two factors including temperature (T) and treatment (Tr) as well as the interaction effect between temperature and treatment (T x Tr) were tested for significance in the two-way PERMANOVA. P-values in bold indicate a significant effect ($p < 0.05$).

Group	Variable	Factor	*df*	SS	MS	Pseudo-F	P (perm)	Unique	P (MC)
Oxygen Fluxes	Net Photosynthesis (P_{net})	Temperature (T)	1	2.79	2.79	4.07	0.044	995	0.055
		Treatment (Tr)	1	1.18	1.18	1.72	0.200	996	0.210
		T x Tr	1	4.59	4.59	6.70	**0.017**	998	**0.019**
		Residuals	19	13.03	0.69				
		Total	22	22.00					
	Respiration (R)	Temperature (T)	1	5.45	5.45	12.89	**0.004**	998	**0.003**
		Treatment (Tr)	1	1.23	1.23	2.91	0.105	996	0.092
		T x Tr	1	6.65	6.65	15.73	**0.001**	996	**0.001**
		Residuals	19	8.03	0.42				
		Total	22	22.00					
	Gross Photosynthesis (P_{gross})	Temperature (T)	1	4.40	4.40	8.71	**0.009**	992	**0.003**
		Treatment (Tr)	1	1.32	1.32	2.60	0.099	996	0.146
		T x Tr	1	6.11	6.11	12.08	**0.001**	996	**0.003**
		Residuals	19	9.61	0.51				
		Total	22	22.00					

Table S6 Results of the two-way PERMANOVA (type III) and Monte-Carlo test for the photosynthetic efficiency and variables derived from the rapid light curves (alpha, ps/ETR$_{max}$, E$_k$) of *Pocillopora verrucosa*. Two factors including temperature (T) and treatment (Tr) as well as the interaction effect between temperature and treatment (T x Tr) were tested for significance. P-values in bold indicate a significant effect ($p < 0.05$).

Group	Variable	Factor	*df*	SS	MS	Pseudo-F	P (perm)	Unique	P (MC)
Photosynthetic Efficiency	*Fv / Fm*	Temperature (T)	1	0.38	0.38	0.35	0.581	991	0.569
		Treatment (Tr)	1	0.23	0.23	0.21	0.608	996	0.634
		T x Tr	1	0.57	0.57	0.52	0.482	992	0.495
		Residuals	20	21.82	1.09				
		Total	23	23.00					
Rapid Light Curves	alpha	Temperature (T)	1	2.91	2.91	2.90	0.105	997	0.093
		Treatment (Tr)	1	0.00	0.00	0.00	0.995	998	0.994
		T x Tr	1	0.03	0.03	0.03	0.875	995	0.882
		Residuals	20	20.06	1.00				
		Total	23	23.00					
	ps / ETR$_{max}$	Temperature (T)	1	2.75	2.75	2.99	0.109	993	0.109
		Treatment (Tr)	1	1.59	1.59	1.73	0.228	997	0.191
		T x Tr	1	0.31	0.31	0.33	0.568	999	0.568
		Residuals	20	18.36	0.92				
		Total	23	23.00					
	E$_k$	Temperature (T)	1	5.30	5.30	6.18	**0.026**	995	**0.018**
		Treatment (Tr)	1	0.47	0.47	0.55	0.460	993	0.489
		T x Tr	1	0.08	0.08	0.10	0.757	997	0.770
		Residuals	20	17.15	0.86				
		Total	23	23.00					

Table S7 Results of the two-way PERMANOVA (type III) and Monte-Carlo test for Symbiodiniaceae cell density and chlorophyll a and c2 content of *Pocillopora verrucosa*. Two factors including temperature (T) and treatment (Tr) as well as the interaction effect between temperature and treatment (T x Tr) were tested for significance. P-values in bold indicate a significant effect ($p < 0.05$).

Group	Variable	Factor	*df*	SS	MS	Pseudo-F	P (perm)	Unique	P (MC)
Symbiodiniaceae Cell Density	Symbiodiniaceae Cell Density	Temperature (T)	1	0.75	0.75	0.82	0.381	996	0.397
		Treatment (Tr)	1	0.79	0.79	0.86	0.365	997	0.365
		T x Tr	1	3.15	3.15	3.44	0.076	997	0.093
		Residuals	20	18.31	0.92				
		Total	23	23.00					
Chlorophyll	Chlorophyll a ($\mu g\ cm^{-2}$)	Temperature (T)	1	1.72	1.72	1.97	0.173	998	0.183
		Treatment (Tr)	1	3.41	3.41	3.91	0.062	997	0.056
		T x Tr	1	0.42	0.42	0.48	0.504	997	0.479
		Residuals	20	17.45	0.87				
		Total	23	23.00					
	Chlorophyll a ($pg\ cell^{-1}$)	Temperature (T)	1	0.40	0.40	0.41	0.534	996	0.509
		Treatment (Tr)	1	0.97	0.97	1.01	0.339	999	0.339
		T x Tr	1	2.50	2.50	2.61	0.116	997	0.123
		Residuals	20	19.14	0.96				
		Total	23	23.00					
	Chlorophyll c2 ($\mu g\ cm^{-2}$)	Temperature (T)	1	0.90	0.90	1.04	0.323	997	0.299
		Treatment (Tr)	1	4.39	4.39	5.08	**0.036**	994	**0.039**
		T x Tr	1	0.40	0.40	0.47	0.489	996	0.481
		Residuals	20	17.31	0.87				
		Total	23	23.00					
	Chlorophyll c2 ($pg\ cell^{-1}$)	Temperature (T)	1	0.00	0.00	0.00	0.967	999	0.965
		Treatment (Tr)	1	1.46	1.46	1.51	0.228	996	0.225
		T x Tr	1	2.21	2.21	2.29	0.146	997	0.149
		Residuals	20	19.33	0.97				
		Total	23	23.00					

References

1. Platt T, Gallegos CL, Harrison WG. Photoinhibition of photosynthesis in natural assemblages of marine phytoplankton. J Mar Res. 1980; 38.
