# Supplementary material for: Temperature-dependent responses of the hard corals Acropora sp. and Pocillopora verrucosa to molecular hydrogen
Source: PLoS One. 2025 Mar 25;20(3):e0308894. doi: 10.1371/journal.pone.0308894 (PMC11936180; doi:10.1371/journal.pone.0308894)
Supplement: Attachment — Submitted filename: Response_to_Reviewers_auresp_2.docx [file pone.0308894.s005.docx]

**Editor:**

Dear Dr. Ostendarp,

Thank you for submitting your manuscript to PLOS ONE. After careful consideration, we feel that it has merit but does not fully meet PLOS ONE’s publication criteria as it currently stands. Therefore, we invite you to submit a revised version of the manuscript that addresses the points raised during the review process.

Please submit your revised manuscript by Jan 31 2025 11:59PM. If you will need more time than this to complete your revisions, please reply to this message or contact the journal office at plosone@plos.org. When you're ready to submit your revision, log on to https://www.editorialmanager.com/pone/ and select the 'Submissions Needing Revision' folder to locate your manuscript file.

Please include the following items when submitting your revised manuscript:

A rebuttal letter that responds to each point raised by the academic editor and reviewer(s). You should upload this letter as a separate file labeled 'Response to Reviewers'.

A marked-up copy of your manuscript that highlights changes made to the original version. You should upload this as a separate file labeled 'Revised Manuscript with Track Changes'.

An unmarked version of your revised paper without tracked changes. You should upload this as a separate file labeled 'Manuscript'.

If you would like to make changes to your financial disclosure, please include your updated statement in your cover letter. Guidelines for resubmitting your figure files are available below the reviewer comments at the end of this letter.

If applicable, we recommend that you deposit your laboratory protocols in protocols.io to enhance the reproducibility of your results. Protocols.io assigns your protocol its own identifier (DOI) so that it can be cited independently in the future. For instructions see: https://journals.plos.org/plosone/s/submission-guidelines#loc-laboratory-protocols.

Additionally, PLOS ONE offers an option for publishing peer-reviewed Lab Protocol articles, which describe protocols hosted on protocols.io. Read more information on sharing protocols at https://plos.org/protocols?utm_medium=editorial-email&utm_source=authorletters&utm_campaign=protocols.

We look forward to receiving your revised manuscript.

Kind regards,

Anderson B. Mayfield, Ph.D.

Academic Editor

PLOS ONE

Journal Requirements:

Please review your reference list to ensure that it is complete and correct. If you have cited papers that have been retracted, please include the rationale for doing so in the manuscript text, or remove these references and replace them with relevant current references. Any changes to the reference list should be mentioned in the rebuttal letter that accompanies your revised manuscript. If you need to cite a retracted article, indicate the article’s retracted status in the References list and also include a citation and full reference for the retraction notice.

Additional Editor Comments:

Thank you for your patience. I have now had this article reviewed by another two reviewers, one of whom endorsed it in full and the second with only some minor comments. As such, I believe this article is nearing the finish line. I would, however, urge you to ensure that all concerns of the very first reviewer from the first round of review were thoroughly addressed, as they were quite severe (pointing to some potentially critical experimental design flaws). If that reviewer's interpretation of the design was correct, then the article may ultimately have a diminished impact. Just a thought since, as someone employing coral reef interventions myself, we want to ensure that these methods are robust before they start spreading life wildfire!

**Our Response:**

Dear Dr. Anderson B. Mayfield,

We would like to resubmit our revised manuscript entitled: “Temperature-dependent responses of the hard corals *Acropora* sp. and *Pocillopora verrucosa* to molecular hydrogen”. We sincerely appreciate the constructive and valuable feedback from yourself and the other two reviewers. We have addressed all the comments, as outlined in the table below, detailing the actions taken and the corresponding changes made to the manuscript. We fully agree with your statement and have included a detailed limitations section in the previous revision, along with removing potential misinterpretations of the ecological implications in this round of revisions.

Minor revisions include updates to the introduction and discussion sections together with the reference list to provide a clearer explanation of the response parameters, particularly regarding coral thermal tolerance and their interdependence. Overall, we believe that the manuscript's clarity, especially concerning the response parameters, has improved, while minimizing potential misunderstandings of the ecological implications.

Yours sincerely on behalf of all co-authors,

Malte Ostendarp

**Comments from “reviewer 4” and author’s response:**

| # | Paragraph / Lines | Comments | Author’s action | Author’s response |
| --- | --- | --- | --- | --- |
| 1 | Introduction | The authors presume that readers are familiar with the terms and biological relevance of various physiological traits connected to photosynthesis and they do not explain or discuss them in the paper. Since I am not an expert on photosynthesis, I found it challenging to follow the results and discussion when terms like P gross, P net, respiration, ETRmax and Ek are discussed within the context of nutrient shuffling and coral-algal symbiosis maintenance. It would enhance understanding if the authors explained what these traits represent as proxies and how their shifts are relevant to the biology of coral thermal resilience. | Changed. | Thanks for this comment. We fully agree and have added a section at the end of the introduction to group the response parameters and explain their meaning in the context of coral thermal resilience (L.86-104). We have also clarified how shifts in these traits, particularly under heat stress, serve as important proxies for changes in coral-algal symbiosis and photosynthetic capacity. |
| 2 | Fig. 3-4 | Figure 3 and Figure 4 labels don’t correspond to their respective mentions in the text. I didn’t find any mention/explication of figure 3A, while figure 4A is mislabeled as ETRmax and 4B as Ek in the text (line 360 in the tracked-changes version). | Changed. | Thank you very much for the comment. We have updated the labels in the text. |
| 3 | Discussion | I am not sure I follow the authors’ deduction that, based on their results, they believe molecular hydrogen prevents coral bleaching through its competition with nitrogen. My understanding is that the coral host limits nitrogen bioavailability to the symbiont to reduce its population growth. Although the doubling time of symbionts in adult coral tissue is debated, the general consensus is that it occurs over days rather than hours, as far as I know (and I might be wrong). So it is already challenging to discuss the effect on population growth based on the experiment that lasted only 48 hours. Moreover, the data presented show that hydrogen addition had no effect on the coral respiration (Fig 1), photosynthetic efficiency (Fig 2), Symbiodiniacae density, or chlorophyll content, but it did affect ETRmax (Fig 3) in the Acropora species. In Pocillopora corals, hydrogen addition affected coral respiration (Fig 1) and chlorophyll content (Figure 5) with no effect on photosynthesis efficiency (Fig 2) or symbiont cell density. It’s two different effects and as much as I understand differences between coral species, I don’t find that there is enough evidence in the discussion right now to claim that these changes are caused by a difference in nitrogen bioavailability to the symbiont. Could authors discuss detailed evidence from other literature that connects these traits to nitrogen availability? How do they reconcile the differences between species while drawing a general model from the data? (Figure 6) | Changed. | Thank you for your feedback. We acknowledge the ongoing debate regarding the generation time of Symbiodiniaceae. Based on studies suggesting a generation time of one to several days, we believe that effects could occur within the 48-hour timeframe of our experiment. For *Pocillopora verrucosa*, we observed significant reductions in photosynthesis and respiration rates, as well as areal chlorophyll c2 content (see Fig. 1 and Fig. 5). Although not statistically significant, the lowest Symbiodiniaceae cell density and areal chlorophyll a content were also recorded for fragments at ambient temperatures exposed to molecular hydrogen, indicating a loss of Symbiodiniaceae cells during this period.  In response to your suggestion, we have expanded our discussion to include the generation time of Symbiodiniaceae and the potential links between the measured parameters and nitrogen availability (L. 438-443). Additionally, we have included a segment addressing the observed differences between *Acropora* sp. and *Pocillopora verrucosa* at ambient temperatures (L. 443-447). These differences may origin from differences in nitrogen fixation rates between the two species, with *Pocillopora verrucosa* potentially having a lower nitrogen fixation rate, resulting in a more pronounced inhibition effect under molecular hydrogen exposure.  Since nitrogen fixation rates were shown to increase under heat stress potentially linked to coral bleaching, we additionally hypothesize that hydrogen counteracts this effect and thereby maintains the nitrogen limitation potentially minimizing negative effects of heat stress although this remains speculative.  Lastly, we want to clarify that the model presented in Fig. 6 is intended to summarize these potential mechanisms of molecular hydrogen under heat stress. We hope that these additions and clarifications effectively address your concerns and improve the interpretation of our findings. |
| 4 | Discussion | On the same note, the authors claim that hydrogen addition presumably prevents/mitigates coral bleaching. However, I do not see evidence in the presented data supporting this statement. Where in the data do they demonstrate that molecular hydrogen mitigates coral bleaching? | Responded. | Thank you for your comment. We based our statement that hydrogen can minimize the negative effects of heat stress on its impact on the maximum electron transport rate (ETR_max_) in *Acropora* sp. (see Fig. 3B) Under heat stress, the addition of molecular hydrogen resulted in an ETR_max_ that was significantly higher by 28% compared to heat stress alone, where ETR_max_ was significantly reduced compared to ambient temperature conditions.  This finding suggests that molecular hydrogen helps mitigate negative effects of temperature-induced stress. While these results do not directly demonstrate reduced coral bleaching (e.g. effects on symbiont density or Chl_a_ and Chl_c2_, see Fig. 5), they highlight a physiological response associated with increased resilience of the coral holobiont under temperature stress.  We agree that further evidence is crucial to confirm the effects of molecular hydrogen on coral bleaching. For this reason, we cautiously state that hydrogen minimizes negative effects of heat stress that could potentially be linked to coral bleaching over the long term thereby providing valuable insights into the potential of molecular hydrogen as a stress antagonist. |
| 5 | Discussion | Furthermore, they discuss the potential strategies on how to use molecular hydrogen on reefs to mitigate damage during acute thermal stress. For example, the sentence “Primarily, molecular hydrogen should only be administered during heat stress events with hydrogen gas concentrations lower than 4% to minimize the risk of explosion…” (line 545) reads like a direct instruction for field application. This is concerning, as there is no definitive proof in this study that molecular hydrogen has an effect on coral thermal resilience or mortality. Additionally, the long-term effects and trade-offs of molecular hydrogen on coral health and reef ecosystems remain unknown but can be detrimental. Suggesting untested interventions could be dangerous, especially if individuals with good intentions but limited expertise act on this advice. I believe scientific community should be very careful about how we communicate our results to a broad audience, particularly when proposing strategies to mitigate reef damage under climate change. Only scientifically validated methods should be proposed with such direct wording in scientific literature. | Changed. | We completely agree, as this sentence could easily be misinterpreted, so we have removed it entirely. We also checked the text and only propose follow-up questions without giving advice on the potential application. |
| 6 | General | Despite these concerns, I commend the authors for highlighting the potential of molecular hydrogen in studying coral-algal symbiosis maintenance and disruption. Understanding the cellular and molecular principles of coral bleaching and resilience is crucial for developing effective reef protection and restoration strategies. Every promising approach should be carefully and comprehensively studied before being proposed for real-world application. The authors offer new and potentially powerful perspectives on coral reef protection, and their work should be published after addressing these points. | Responded. | Thank you very much for your constructive and thorough review! We agree to all points you made and hope we could address your comments and concerns within the manuscript. |

**Comments from “reviewer 5” and author’s response:**

| # | Paragraph / Lines | Comments | Author’s action | Author’s response |
| --- | --- | --- | --- | --- |
| 1 | General | This manuscript aims to address a key outstanding question in coral ecophysiology of the cellular mechanisms of coral bleaching and offers insights into theoretical interventions for bleaching-mitigation. I commend the simultaneous use of multiple methods including algal symbiont flow cytometry, quantification of symbiont chlorophyll content, measuring photochemical efficiency and quantifying photosynthesis:respiration ratios to assess holobiont heat tolerance. Together, results provide multiple complementary lines of evidence to assess the interacting effects of short-term heat stress and molecular hydrogen on photosynthetic & photochemical efficiency in the coral holobiont. I agree with previous reviewers that re-framing the manuscript around the temperature-dependence of the effect of hydrogen on the heat stress response better supports the results reported and has improved the manuscript. The manuscript is generally logically organised, with detailed and thorough descriptions of most methods and clear figures which are intuitive to interpret and aesthetically consistent throughout the manuscript. I would recommend the addition of the following to the introduction and discussion sections to prepare this manuscript for publication: Firstly, the relevance of the metrics reported in the results to the ‘temperature tolerance’ of the holobionts needs to be prefaced in the introduction before the reader can relate results to the hypothesis. Secondly, additional summarising of results is needed in the discussion to more comprehensively interpret the nuances of how heat and hydrogen affected various facets of photochemical performance in each species tested. | Responded. | Thank you very much for your constructive and thorough review of our manuscript! We hope that we could address your comments and concerns within the revised version of the manuscript. |
| 2 | L. 57 | More detail on the oxidative theory of coral bleaching would be helpful here. As would brief mention of other prominent mechanistic theories for coral bleaching. eg the shift from nitrogen-limitation to carbon-limitation (especially since you address nitrogen limitation in your discussion). | Changed. | We fully agree and have expanded on the details of the oxidative theory, while also highlighting the role of nitrogen limitation in coral bleaching (L. 56-63). |
| 3 | L. 76 | The final paragraph in the introduction would benefit from more setup to prepare the reader for the results they’re about to see (specifically, the relationships between photosynthesis, respiration, photochemical efficiency, ETRmax, Ek and chlorophyll, and how these metrics relate to ‘temperature tolerance’). | Changed. | Thanks for your feedback. We have added a paragraph at the end of the introduction that groups all the parameters under the categories of bleaching phenotype, photosynthetic capacity and holobiont phenotype (L. 86-104). Additionally, we gave a brief explanation of each parameter and their relevance to heat stress and heat tolerance. |
| 4 | L. 97 | Since fragments for molecular hydrogen treatments were collected from your colonies after fragments for control treatments, it’s possible that the wound-healing responses triggered by earlier collections affected coral responses to hydrogen treatments- worth addressing this somewhere. | Responded. | The collection dates for each treatment were assigned randomly, starting with H_2_26, then CT32 and H_2_32, and ending with CT26 (see supplementary file), so not only the hydrogen treatment should have been affected. Since all colonies were also used in a previous experiment one month earlier, the wound healing process should have impacted all treatments equally. |
| 5 | L. 133 | In light of the unintended temperature variation, it would be helpful to see the per-treatment temperature profiles visualised in your supplemental materials. | Responded. | We consistently monitored the temperature throughout the day and set up an alarm to detect deviations exceeding 0.5 °C from the target temperature. Unfortunately, the malfunction occurred at night, and we only detected the malfunction of the heater in the early morning, meaning it happened during a 12-hour window. Aside from this incident, we ensured the temperature remained within the intended range by regularly checking the temperature controller. We agree that this would be helpful, however, since we measured the temperature only once per day with a handheld sensor to check the display of the temperature controller, we believe it is more appropriate to provide a temperature range given in the M&M section rather than a temperature profile based on the three data points from the handheld sensor. |
|  | L. 228 | How did you account for between-colony differences across these results? i.e. did you include colony effects in models? | Responded. | We were unable to account for colony effects within the models as we only had biological but not technical replicates meaning we had six fragments, one fragment from each of the six colonies, for every parameter. We chose this design to account for natural variability but due to space and time restrictions, we were unable to have both biological and technical replicates. |
| 7 | L. 268 | No explanation in the methods for why several colony replicates were excluded from two of the treatments. | Changed. | Thanks for the notice. We have included that these three measurements had to be excluded due to a measurement error (L. 295). |
| 8 | L. 353 | At the start of the discussion, it would be helpful to have a summary of the main results and how the different metrics complement/oppose each-other with respect to the effects of heat and hydrogen on temperature tolerance in these two species. | Responded. | We have included a summary of all the parameters, particularly in relation to heat stress, at the end of the introduction. Additionally, at the beginning of each research question within the discussion, we provided an overview of each affected parameter. We believe that explaining each parameter in detail again at this point may make the section more complex to understand. |
| 9 | L. 379 | Lacking some comment on possible reasons why Acropora & Pocillopora responses differed. | Changed. | We have added a section discussing potential reasons why hydrogen has mainly affected *Pocillopora verrucosa* under ambient temperatures (L. 443-447) and why heat stress might have mainly affected *Acropora* sp. (L. 411-414). |
| 10 | L. 416 | Comment on symbiont taxa commonly associated with your study species in reefs near to your collection site, since this would surely affect the metrics recorded in this study relating to photosynthesis. | Changed. | We have included the dominant symbiont taxa for both taxa which are commonly associated with both species in the Red Sea and included it in the discussion in the explanation why both species might have responded differently to heat stress (L.411-414). |
