# Supplementary material for: Temperature-dependent responses of the hard corals Acropora sp. and Pocillopora verrucosa to molecular hydrogen
Source: PLoS One. 2025 Mar 25;20(3):e0308894. doi: 10.1371/journal.pone.0308894 (PMC11936180; doi:10.1371/journal.pone.0308894)
Supplement: Attachment — Submitted filename: Response to Reviewers.docx [file pone.0308894.s004.docx]

**Editor:**

Dear Dr. Ostendarp,

Thank you for submitting your manuscript to PLOS ONE. After careful consideration, we feel that it has merit but does not fully meet PLOS ONE’s publication criteria as it currently stands. Therefore, we invite you to submit a revised version of the manuscript that addresses the points raised during the review process.

Please submit your revised manuscript by Nov 10 2024 11:59PM. If you will need more time than this to complete your revisions, please reply to this message or contact the journal office at [plosone@plos.org](mailto:plosone@plos.org). When you're ready to submit your revision, log on to <https://www.editorialmanager.com/pone/> and select the 'Submissions Needing Revision' folder to locate your manuscript file.

Please include the following items when submitting your revised manuscript:

- A rebuttal letter that responds to each point raised by the academic editor and reviewer(s). You should upload this letter as a separate file labeled 'Response to Reviewers'.
- A marked-up copy of your manuscript that highlights changes made to the original version. You should upload this as a separate file labeled 'Revised Manuscript with Track Changes'.
- An unmarked version of your revised paper without tracked changes. You should upload this as a separate file labeled 'Manuscript'.

If you would like to make changes to your financial disclosure, please include your updated statement in your cover letter. Guidelines for resubmitting your figure files are available below the reviewer comments at the end of this letter.

If applicable, we recommend that you deposit your laboratory protocols in [protocols.io](http://protocols.io/) to enhance the reproducibility of your results. Protocols.io assigns your protocol its own identifier (DOI) so that it can be cited independently in the future. For instructions see: [https://journals.plos.org/plosone/s/submission-guidelines#loc-laboratory-protocols](https://c05y1x9s.r.us-east-2.awstrack.me/L0/https:%2F%2Fjournals.plos.org%2Fplosone%2Fs%2Fsubmission-guidelines%23loc-laboratory-protocols/1/010f01922cd11964-203be66b-af07-466a-89ec-d468c1e02b9d-000000/LDLfImK-GXpYMoCD80bVH69Nm9g=178). Additionally, PLOS ONE offers an option for publishing peer-reviewed Lab Protocol articles, which describe protocols hosted on [protocols.io](http://protocols.io/). Read more information on sharing protocols at [https://plos.org/protocols?utm_medium=editorial-email&utm_source=authorletters&utm_campaign=protocols](https://c05y1x9s.r.us-east-2.awstrack.me/L0/https:%2F%2Fplos.org%2Fprotocols%3Futm_medium=editorial-email%26utm_source=authorletters%26utm_campaign=protocols/1/010f01922cd11964-203be66b-af07-466a-89ec-d468c1e02b9d-000000/ce_fwMvwkTGFoudCJzxAlUFmFos=178).

We look forward to receiving your revised manuscript.

Kind regards,

Anderson B. Mayfield, Ph.D.
Academic Editor
PLOS ONE

Journal Requirements:

When submitting your revision, we need you to address these additional requirements.

1. Please ensure that your manuscript meets PLOS ONE's style requirements, including those for file naming. The PLOS ONE style templates can be found at

<https://journals.plos.org/plosone/s/file?id=wjVg/PLOSOne_formatting_sample_main_body.pdf> and 
<https://journals.plos.org/plosone/s/file?id=ba62/PLOSOne_formatting_sample_title_authors_affiliations.pdf>

2. We note that the grant information you provided in the ‘Funding Information’ and ‘Financial Disclosure’ sections do not match.

When you resubmit, please ensure that you provide the correct grant numbers for the awards you received for your study in the ‘Funding Information’ section.

3. Please expand the acronym “KAUST” (as indicated in your financial disclosure) so that it states the name of your funders in full.

This information should be included in your cover letter; we will change the online submission form on your behalf.

4. Please note that funding information should not appear in the Acknowledgments section or other areas of your manuscript. We will only publish funding information present in the Funding Statement section of the online submission form. Please remove any funding-related text from the manuscript.

5. When completing the data availability statement of the submission form, you indicated that you will make your data available on acceptance. We strongly recommend all authors decide on a data sharing plan before acceptance, as the process can be lengthy and hold up publication timelines. Please note that, though access restrictions are acceptable now, your entire data will need to be made freely accessible if your manuscript is accepted for publication. This policy applies to all data except where public deposition would breach compliance with the protocol approved by your research ethics board. If you are unable to adhere to our open data policy, please kindly revise your statement to explain your reasoning and we will seek the editor's input on an exemption. Please be assured that, once you have provided your new statement, the assessment of your exemption will not hold up the peer review process.

**Additional Editor Comments:**

Hello,

Thank you for submitting your article to PLoS ONE, and I apologize for the length of time it took to have it reviewed. All three reviewers appreciated the novelty of the idea but had issues with the experimental design and interpretation of the data, especially given the overall small scope of the project; the conclusions, then, may not be commensurate with the results (some of which do NOT support the prevailing primary conclusion). One reviewer recommended rejection, but I am hoping that some of the experimental design issues were simply described inadequately. If, however, this reviewer (#1) is correct in their interpretation of how the study was conducted, I may instead recommend that further experimentation be performed, with a later resubmission. As such, I am leaving this open as a "major revision," but if you do not think you can address the reviewers' concerns, I completely understand your decision to either re-submit after further data are collected (or trying your luck with another journal). Hopefully, at a minimum, you find the reviewers' comments useful, and we certainly need new means of fostering coral resilience, either through environmental improvement or boosting of thermotolerance, and so the novel nature of the study is certainly commendable.

**Our Response:**

Dear Dr. Anderson B. Mayfield,

We would like to resubmit our revised manuscript entitled: “Temperature-dependent responses of the hard corals *Acropora* sp. and *Pocillopora verrucosa* to molecular hydrogen”. We sincerely appreciate the constructive and valuable feedback from the three reviewers and yourself. We have addressed all these comments in the table below, highlighting our actions and the corresponding changes made in the manuscript.

Major changes include the revision of the title, abstract, results and discussion section to more accurately reflect the temperature-dependent effects of molecular hydrogen adjusting the overall conclusion. Additionally, we have provided a statement regarding the crucial concerns of the experimental design justifying our analysis, while also clearly stating these limitations within the manuscript. Overall, we believe that both the clarity and coherence of the entire manuscript have been enhanced, resulting in a more comprehensive presentation of our findings.

Yours sincerely on behalf of all co-authors,

Malte Ostendarp

**Comments from editor and author’s response:**

| # | Paragraph / Lines | Comments | Author’s action | Author’s reponse |
| --- | --- | --- | --- | --- |
| 1 | General | Please ensure that your manuscript meets PLOS ONE's style requirements, including those for file naming. | Responded. | We have rechecked our manuscript style according to PLOS ONE’s style requirements. |
| 2 | Financial Disclosure | We note that the grant information you provided in the ‘Funding Information’ and ‘Financial Disclosure’ sections do not match.  When you resubmit, please ensure that you provide the correct grant numbers for the awards you received for your study in the ‘Funding Information’ section. | Responded. | Thank you for the comment, we have verified the grant numbers in the Funding Information section |
| 3 | Financial Disclosure | Please expand the acronym “KAUST” (as indicated in your financial disclosure) so that it states the name of your funders in full.  This information should be included in your cover letter; we will change the online submission form on your behalf. | Responded/  Changed. | We expanded the acronym “KAUST” in the manuscript. Unfortunately, we were not able to access and change the financial disclosure on the review platform. Thank you for adjusting the online submission form! |
| 4 | Acknow-  legements | Please note that funding information should not appear in the Acknowledgments section or other areas of your manuscript. We will only publish funding information present in the Funding Statement section of the online submission form. Please remove any funding-related text from the manuscript. | Changed. | We removed any funding-related text from the manuscript. |
| 5 | Data availability | When completing the data availability statement of the submission form, you indicated that you will make your data available on acceptance. We strongly recommend all authors decide on a data sharing plan before acceptance, as the process can be lengthy and hold up publication timelines. Please note that, though access restrictions are acceptable now, your entire data will need to be made freely accessible if your manuscript is accepted for publication. This policy applies to all data except where public deposition would breach compliance with the protocol approved by your research ethics board. If you are unable to adhere to our open data policy, please kindly revise your statement to explain your reasoning and we will seek the editor's input on an exemption. Please be assured that, once you have provided your new statement, the assessment of your exemption will not hold up the peer review process. | Responded. | We have already uploaded our data with the manuscript and would like to make the data publicly available within the supplementary material of the manuscript. |

**Comments from “reviewer 1” and author’s response:**

| # | Paragraph / Lines | Comments | Author’s action | Author’s response |
| --- | --- | --- | --- | --- |
| 1 | General | This manuscript describes intriguing findings with regard to molecular hydrogen mitigating heat stress in Acropora sp. corals. However, I have some fundamental concern regarding the experimental design; specifically lack of replication. As I understand (and perhaps I have misunderstood), each of the four treatments was applied on a separate day, to a separate collection of fragments, in a single aquarium. In this case, although there were six colonies involved, these represent six biological samples, but they are not statistical replicates because they do not represent an independent application of the treatment (the treatments were applied in a single tank). Hence, the application of inferential statistics is not valid (Hurlburt 1984 Ecol Monog 54: 187-211). | Responded. | Thank you very much for your valuable review and for raising this very important concern regarding our experimental design, particularly the pseudoreplication. We acknowledge that using a single tank for each treatment and species represents a limitation of this study. Our decision for this experimental setup was driven by the feasibility of treatment application, the need for controlled environmental conditions, and the aim to capture a large biological variability of corals. Given these constraints, our experimental setup was restricted to one tank per treatment and species as it was designed to apply the respective treatment and heat stress within 48 h rather than for long-term coral maintenance.  Notably, this approach aligns with several other short-term coral studies, specifically those involving the Coral Bleaching Automated Stress System (CBASS), which served as a foundation for our work. In these studies, coral fragments are subjected to controlled short-term heat stress treatments within a single tank for each temperature regime to ensure consistent environmental conditions. Similar to our work, these experiments are designed to evaluate immediate physiological responses to heat stress, while including the addition of treatments rather than to allow for an extended maintenance of coral fragments (Alderdice et al. 2022, doi: 10.1038/s41598-022-22604-3; Voolstra et al. 2021, doi: 10.1111/mec.16064; Alderdice et al. 2024, doi: 10.1007/s00338-024-02505-9; Cunning et al. 2024, doi: 10.1007/s00338-024-02577-7). While our design may not achieve full statistical independence as outlined by Hurlbert (1984), we believe it remains effective for assessing physiological responses over a short duration (48 h).  Furthermore, previous studies have demonstrated that maintaining corals in controlled aquarium conditions does not alter their microbiome composition or activity over several weeks, as shown for *Pocillopora verrucosa* (Pogoreutz et al. 2017, doi: 10.1111/gcb.13695).  To mitigate any potential tank effects, we therefore ensured that our experimental conditions (e.g. temperature, salinity, light and flow rate) were strictly controlled and monitored in all our tanks. To further account for a larger biological variability, we decided to use six fragments from six different colonies. We therefore believe that our study characterizes short-term effects of molecular hydrogen on the coral holobiont and, as such, offers a valuable contribution to the current literature.  We have now added the abovementioned justification of our experimental design to the method section (“Our decision for this approach was driven by the need for controlled environmental conditions, the feasibility of treatment application, and the aim to capture a large biological variability of corals. Consequently, our setup was restricted to one tank per treatment and species, as it was intended to apply heat stress and treatments over a 48-h period rather than for extended coral maintenance.”). In addition, we added a new section to the discussion that includes the abovementioned limitations and related explanations (“It is, however, important to acknowledge the study's limitations. The effects of molecular hydrogen were examined in only two hard coral species in the Central Red Sea and were limited to 48 h. Additionally, the experimental setup utilized a single tank for each treatment and species, designed specifically for treatment application rather than extended coral maintenance. While this design allowed precise and consistent environmental conditions and treatment addition, future long-term studies should aim to include an acclimatization phase and a greater number of tanks to mitigate potential tank effects over extended periods. Despite these limitations, our research offers initial evidence of the temperature-dependent effects of molecular hydrogen. Therefore, it can pave the way for future studies especially investigating following research questions: What specific mechanisms underlie molecular hydrogen's role in coral holobionts, particularly in relation to nitrogen fixation and oxidative damage (Hypothesized mechanisms in Fig. 6)? What are the long-term effects of molecular hydrogen on coral holobionts under both ambient and heat-stressed conditions?”). |
| 2 | Materials & Methods | Meanwhile, I am not a photophysiologist, so cannot comment on the appropriateness of the photophysiological methods/calculations/models. | Responded. | The photophysiological assessments were used as an indicator for the stress state of the endosymbionts and the calculations were done according to studies from Ralph and Gademann (2005, doi: 10.1016/j.aquabot.2005.02.006) and Ralph et al. (2005, doi:10.1111/j.1529-8817.2005.04034.x) using similar models and calculations already described (Platt et al. 1980). |
| 3 | General | The target, responsive species in the study is referred to as Acropora spp. Firstly, spp. is a plural notation (i.e. it refers to multiple species). If the authors purport that this sample is a single species (some defense of this point could be appropriate), the correct notation would be ‘sp.’ I understand that Red Sea Acropora may have particular taxonomic challenges, but can any identifying information be given with regard to this responsive sp.? (e.g. growth form, photo,??). Relatedly, since you have tested only one (unnamed) Acropora spp. you should refrain from extrapolating to the whole genus in the title. | Changed. | *Acropora* colonies were chosen on the basis of morphological characteristics that resemble *Acropora* *hemprichii*. We have therefore changed *Acropora* spp. to A*cropora* sp. throughout the manuscript and included this information in the Materials & Methods section: “*Acropora* fragments were collected based on morphological characteristics (*Acropora* cf. *hemprichii*). However, due to the complex taxonomy within the genus *Acropora*, we decided to conservatively refer to *Acropora* sp.” The title was replaced and adjusted accordingly. |
| 4 | Results | Lastly, I found the presentation of the results extremely difficult to follow. Generally, results are presented in a parallel order with the methods and a single statistical test/model is described at the time. I appreciate that the authors are attempting to link the different responses in the results, but it was very hard to follow. Better to order the results talking about one response/test at the time and leave the linkage for the discussion. | Changed. | Thank you for this suggestion. We have rewritten and restructured the results accordingly. |
| 5 | L. 111 | is it possible to do any validation of the manufacturer’s claim in regard to the actual H2 levels? I do not understand the chemistry of how these ‘generators’ work but I am curious how reliable they are . . . . | Responded. | We contacted the company and they provided us with a test report for this specific hydrogen water generator (attached at the end of this document). |
| 6 | L. 138-139 | Seems nonsensical to call something dead when it is not dead (i.e. 25% alive). As best I can see, the cited Casey et al. 2015 defined it this way “low mortality was defined as 0–20% loss of tissue from the coral branch, partial mortality was defined as 20–80% loss of tissue from the coral branch, and high mortality was defined as 80–99% loss of tissue from the coral branch” (Casey et al. 2015) | Changed. | We agree with this point and changed our definition of survival accordingly (“Survival and bleaching of all fragments were visually assessed directly after the 48 h incubations. Fragments were classified as dead if 100 % tissue loss was visible.”). |
| 7 | Fig. 1 | Confused as to the overlapping bars. I expect the lower portion ® to be the same extent as the non-overlapping portion of the (Pgross) upper bar; but this does not seem to be the case for the first bar (Acropora, Control, 26o). Is this an error? Also, very confusing to interpret statistical results from this graph, and these statistical results in the text are scattered among different sections. The fig captions says “A significant temperature main effect according to the PERMANOVA with Monte-Carlo test (p < 0.05) is presented with letters (above bar: Pgross, below bar: R) and black lines grouping the temperature conditions” However, I do not see any letters below in panel B, and there are two letters (a and b) depicted above the 26o treatment bars. The text ( Ln 274-276) indicates “Ek was the only parameter to exhibit a significant decrease in both control and hydrogen treatments” implying that R and P (Fig 1B) were not significant. I suggest to include the main effect p-values with the figure rather than rely on lines and letters in such a complicated figure. | Changed. | Yes, this is an error, thank you very much for your comment. We had to remove two measurements for *Acropora* sp. CT26 due to a technical defect and these were unfortunately still included in the bar, these are now removed and the reduced sample size for this treatment and species was noted in the figure caption.  We tried to include the main effect p-values with the figure, however, this made the figure more complicated due to the many significant main and interactive effects for all parameters (R, Pnet and Pgross) and therefore we chose to adjust the figure design and figure caption to better visualize the statistical results especially differentiating between panel A and B:  “**Fig. 1 Photosynthesis and respiration rates of (A) *Acropora*** sp. **and (B) *P. verrucosa* for the four groups.** Mean net and gross photosynthesis (P_net_ and P_gross_) and respiration (R) rates are visualized by bars with error bars indicating the respective standard error (SE) of six replica per group (except for four in the CT26 group *Acropora* sp. and five in the CT32 group *P. verruocsa*). The color of the bars indicates the temperature condition (blue = 26 °C, red = 32 °C). For *Acropora* sp. (A), a significant temperature main effect according to the PERMANOVA with Monte-Carlo test is presented with letters (^a/b^ p < 0.05; above bar: P_gross_, below bar: R) and black lines grouping the temperature conditions. For *P. verrucosa* (B), pairwise PERMANOVAs with Monte-Carlo tests were conducted as the interaction effect between temperature and treatment was significant. Significant differences in between treatments are illustrated with asterisks (* p < 0.05, ** p < 0.01; above bar: P_gross_, below bar: R). Exact p-values are presented in supplementary material (S1 File). Coral illustrations were created with BioRender.com.” |
| 8 | Results | The figures are referenced out of order in the text (part of why the results are confusing) | Changed. | Results were rewritten and structured according to the response parameters. The figures are in the right order in the text now. |

**Comments from “reviewer 2” and author’s response:**

| # | Paragraph / Lines | Comments | Author’s action | Author’s response |
| --- | --- | --- | --- | --- |
| 1 | General | The effects observed with these experiments were somewhat small and the story appears complicated in a way that could be more meaningfully addressed in the discussion. Specifically, addressing the results of each of the measured parameters and why these may have deviated from expectations given the proposed mechanisms of hydrogen enrichment that are offered in the introduction. The discussion also spends too much space discussing potential implementation of molecular hydrogen and not enough discussing the results in detail, in the context of the hypothesized function and mechanisms, and how that might interact with environmental variation in situ. Specific future studies should be proposed to fill in some of the gaps of this work, and build on it. | Changed. | We have revised the results and discussion section to clarify the main narrative. The updated version addresses each parameter's outcomes and explores deviations from our initial expectations. The implementation segment was shortened and instead we added a discussion about how molecular hydrogen could affect environmental variation in situ especially regarding other bleaching triggers like cold stress, bacterial infection, light stress and high nitrate concentrations. Additionally, we have included a limitations section and proposed research questions to guide future studies. |
| 2 | General | I would definitely prefer that the experiments ran longer to capture more potential effects of the treatments, with acclimation periods to account for variations in collection timing (or controlling for these analytically, at a minimum), given the incredible sensitivity of these physiological parameters to the environment. That said, these experiments are difficult, labor-intensive, and often logistically-constrained, and I do think the contribution is valuable, if imperfect. | Responded. | We totally agree and are currently designing long-term experiments including acclimation periods. In general, we changed the manuscript to address the limitations of this study clearly. However, we also believe that this study can build the foundation for subsequent studies especially investigating the mechanisms and long-term effects of molecular hydrogen on coral holobionts. |
| 3 | General | The paper would benefit from technical editing to correct typos, grammatical errors, informal language, acronyms that aren’t introduced, and for clarity and completeness. Choosing standardized language for describing the different controls and treatments would help with clarity in the results and discussion sections. | Changed. | The manuscript was proofread by a native English speaker to correct any typos, grammatical errors, informal language and acronyms. The results and discussion part were rewritten including standardized language. |
| 4 | General / Discussion | The specific conclusions are not clear in the discussion, and the conclusion that ‘molecular nitrogen helps corals’ is generalized too broadly there and in the title and abstract. The results only apply to a single species, in the region of the Central Red Sea, and only for one parameter, with a somewhat weak effect. The conclusion should be stated clearly, with clearly delineated assumptions and qualifications about what it might mean, and what limitations the study has for broader interpretation. The explanation related to nitrogen fixation for the difference between hydrogen enrichment on ambient and heat stressed corals makes sense, but is pure speculation and should be identified as such. | Changed. | We revised the discussion, title, and abstract to better reflect the temperature-dependent effects of molecular hydrogen on *Acropora* sp. and *P. verrucosa*. A limitations section was added in the discussion to support a more nuanced interpretation of our findings. Additionally, we shortened the explanation regarding nitrogen fixation, emphasizing that it remains speculative. |
| 5 | General | I recommend technical editing and a rewrite of the discussion to more completely analyze the results and place them in the context of coral ecophysiology. | Changed. | Thank you very much for your thorough review! The manuscript has been technically edited, with the discussion and results sections rewritten to more effectively analyze and interpret findings while clearly outlining the study's limitations. |

**Comments from “reviewer 3” and author’s response:**

| # | Paragraph / Lines | Comments | Author’s action | Author’s response |
| --- | --- | --- | --- | --- |
| 1 | General | My chief concern about this manuscript is that the overall framing does not adequately capture the data. The title, abstract, and summary model figure (Fig. 6) highlight the heat-mitigatory effects of molecular H but neglect to mention that molecular H seems to decrease photosynthetic health (decreased Ek and ETRmax in Acropora, and lower P and R rates in P. verrucosa) at ambient temperatures. These temperature-dependent responses strike me as fundamental results that are worth highlighting in the abstract and title, as they provide critical context and raise biologically interesting questions – i.e., is there a tradeoff whereby the protective effect of molecular H comes at the cost of dampened photophysiology under ambient conditions? If so, it is imperative that any future study of molecular H consider the potential drawbacks of using this to protect corals. As a result, it feels like claims that molecular H “strengthens” coral physiology (line 412) are too generalized. | Changed. | We completely agree and have revised the title, abstract and discussion to emphasize the negative effects of molecular hydrogen at ambient seawater temperature, particularly mentioning the temperature-dependence. Additionally, a limitation section has been included in the discussion to enable a better assessment of the results and their interpretations. |
| 2 | Materials & Methods | Also, actual molecular hydrogen concentrations from the experiment should have been measured and reported with salinity and temperature, since [H2] was the water parameter being manipulated. I understand from the methods as written that the hydrogen water generator manufacturer estimates ~3ppm per cycle for this equipment. However, we don’t know how stable the authors expect that to be during incubation. The concentration could presumably fluctuate based on offgassing, coral or microbial metabolism in the treatment, etc. For the sake of replicability, readers should know the actual [H2]. If these data aren’t currently available, could an additional trial be run with these same [H2] water generators using similar corals under the same conditions to give a sense of the actual incubation [H2]? Can it be estimated somehow? Without being certain whether and to what degree the focal manipulation of this experiment succeeded, it is difficult to put much stock in the conclusions here. | Responded. | We completely agree that this was a limitation of this study. Unfortunately, it is quite complicated to measure dissolved hydrogen and therefore we chose to only include a hydrogen spike within the experiment, where we can only assume the maximum concentration at the addition of the treatment. To make this clearer in the manuscript, we added: “However, as molecular hydrogen is barely soluble [27] and is constantly released, we can only assume a hydrogen spike at the start and after 24 h of the experiment.” We also contacted the company and they provided us with a test report for this specific hydrogen water generator (attached at the end of this document). |
| 3 | Fig. 1 and 2 | The figures are well-organized and clear, particularly the creative and intuitive display of photosynthetic rate parameters in Figure 1. I always prefer to see individual datapoints layered onto box-and-whisker plots to give readers a better sense of the data distribution, so I would recommend that the authors add these to Figures 1 and 2. | Changed. | Thank you very much for the suggestion. We added individual datapoints to Fig. 2 and Fig. 5. However, for Fig. 1 we decided to not include individual datapoints as one bar includes three parameters (Pnet, Pgross and R) and therefore the points seemed very chaotic. |
| 4 | Discussion | Much of the discussion section about nitrogen limitation is interesting but speculative (lines 374-406), and should be condensed. | Changed. | We have condensed this part and emphasized that this part remains speculative. |
| 5 | General | If these concerns can be addressed, it is an intriguing result that molecular H can decrease some bleaching signatures under heat stress in Acropora. The conservation applications of these results are likely limited given the difficulty of distributing molecular hydrogen to wild corals covered in the discussion. I find these data more interesting from a mechanistic perspective, as they might provide additional support for an oxidative-stress-centered model of coral bleaching. I think that with a clearer exploration of the temperature-by-treatment interactions observed here, and a more moderated conclusion about potential benefits of molecular H, this paper could be a valuable contribution to the bleaching literature. | Changed. | Thank you very much for your insightful review! We have made revisions to the manuscript based on your feedback, including a section addressing the mechanistic perspective (“If the hypothesized mechanisms of molecular hydrogen’s effects on the coral holobiont are verified, it could offer invaluable insights into the mechanisms underlying coral bleaching potentially supporting the "Oxidative Theory" [21, 22].”). Additionally, we have revised the overall conclusion of our manuscript to reflect the temperature-dependent effects of molecular hydrogen on both *Acropora* sp. and *P. verrucosa*. |
